# Validation of a post-hypomethylating agent failure prognostic model in myelodysplastic syndromes patients treated in a randomized controlled phase III trial of rigosertib vs. best supportive care

**DOI:** 10.1038/s41408-017-0018-7

**Published:** 2017-12-14

**Authors:** Aziz Nazha, Mikkael A. Sekeres, Rami Komrokji, David P. Steensma, Hagop Kantarjian, Gail Roboz, Pierre Fenaux, Thomas Prebet, Nozar Azarnia, Patrick S. Zbyszewski, Steven M. Fruchtman, Valeria Santini, Lewis R. Silverman, Uwe Platzbecker, Guillermo Garcia-Manero

**Affiliations:** 1 Leukemia Program, Cleveland Clinic Taussig Cancer Institute, Cleveland, OH USA; 2 Department of Malignant Hematology, H. Lee Moffitt Cancer Center & Research Institute, Tampa, FL USA; 3 Department of Medical Oncology, Dana-Farber Cancer Institute, Harvard Medical School, Boston, MA USA; 4 Department of Leukemia, The University of Texas MD Anderson Cancer Center, Houston, TX USA; 5 Division of Hematology and Oncology, New York Presbyterian Hospital-Weill Cornell Medical College, New York, NY USA; 6 Groupe Francophone des mylodysplasies and Service Hematologie Senior, Hopital Saint Louis, Paris, France; 7 Smilow Cancer Center at Yale New Haven Hospital, New Haven, CT USA; 8 Onconova Therapeutics, Inc., Newtown, PA USA; 9 AOU Careggi, University of Florence, Florence, Italy; 10 Division of Hematology/Oncology, Icahn School of Medicine at Mount Sinai, New York, NY USA; 11 Universitätsklinikum Dresden, Dresden, Germany

The hypomethylating agents (HMAs) azacitidine and decitabine are standard therapies for patients with higher-risk myelodysplastic syndromes (MDS)^1, 2^. Although treatment with these agents may improve overall survival (OS), eventually every patient becomes refractory to or relapses after treatment with HMAs, and has a dismal outcome^3^. Clinical trials enrolling patients at the time of HMA failure (HMAF) have often used the International Prognostic Scoring System (IPSS) or the Revised IPSS (IPSS-R) as eligibility criterion for trial entry^4, 5^. We have previously shown that the IPSS, IPSS-R and other commonly used MDS prognostic models (e.g., the World Health Organization classification-based Prognostic Scoring System, and the MD Anderson Prognostic Scoring System) have limited predictive power in the HMAF setting^6^. We therefore developed and validated a new model to predict outcome of patients after HMAF^6^ that includes six clinical variables: age; performance status; complex cytogenetics (>4 abnormalities); marrow blast percentage >20%; platelet count; and red cell transfusion dependency^6^. This model separates patients into two risk categories: lower-risk, with a median OS of 11.0 months (95% confidence interval (CI) 8.8–13.6); and higher-risk disease, with median OS of 4.5 months (95% CI 3.9–5.3)^6^. The model was subsequently validated in an independent cohort of 223 MDS patients derived from the Groupe Francophone des Myélodysplasies database^7^. In the validation cohort, 82 patients were classified in the lower-risk category and they had a median OS of 13 months compared to 141 higher-risk category patients with a median OS of 5 months (*p* < 0.001)^7^.

In this analysis, we validate the model in a cohort of MDS patients treated prospectively on the ONTIME trial, a phase III, randomized clinical trial that evaluated the efficacy of rigosertib vs. best supportive care (BSC) in patients with IPSS intermediate-2 and high-risk MDS assessed after HMAF^8^.

Primary clinical data were obtained from MDS patients who enrolled on the trial. The ONTIME trial is a randomized, controlled phase III trial that enrolled patients with refractory anemia with excess blasts (RAEB)-1, RAEB-2, RAEB-t, and chronic myelomonocytic leukemia who failed treatment with HMA. Patients were randomly assigned (2:1) to receive rigosertib 1800 mg per 24 h via 72-h continuous intravenous infusion administered every other week or BSC with or without low-dose cytarabine^8^. The primary outcome was OS in the intention-to-treat population. HMAF was defined as failure to achieve a response following at least six cycles of azacitidine or at least four cycles of decitabine; relapse after achieving a response (complete remission, partial remission, or hematologic improvement defined by the 2006 International Working Group Criteria), or intolerance to azacitdine or decitabine^8^. OS was calculated from time of randomization to death or last follow-up. Survival curves were constructed using Kaplan–Meier method and compared using log-rank test. Two-sided *p* < 0.05 was considered statistically significant.

Of 299 patients enrolled, 199 received rigosertib and 100 received BSC^8^. The two treatment arms were well balanced in baseline clinical characteristics; 184 patients (62%) had primary HMAF and 115 (38%) had secondary failure. One patient (1%) had low-risk disease per IPSS-R, 14 (7%) intermediate, 67 (34%) high, 93 (47%) very high, and 24 (12%) unknown vs. 0, 14 (14%), 26 (26%), 41 (41%), and 19 (19%) in the BSC arm^8^. With a median follow-up of 19.5 months (interquartile range 11.9–27.3), the median OS was 8.2 months in the rigosertib arm and 5.9 months in the BSC group (hazard ratio (HR) 0.87, 95% CI 0.67–1.14, *p* = 0.33). In a pre-planned exploratory analysis, patients who had primary hypomethylating drug failure in the rigosertib group had a median OS of 8.6 vs. 5.3 months in the BSC group (HR 0.72 (99% CI 0.46–1.13); *p* = 0.06). In the pre-planned analysis of OS, shorter time since diagnosis and lower platelet count were associated with shorter OS irrespective of the treatment group.

**Fig. 1:**
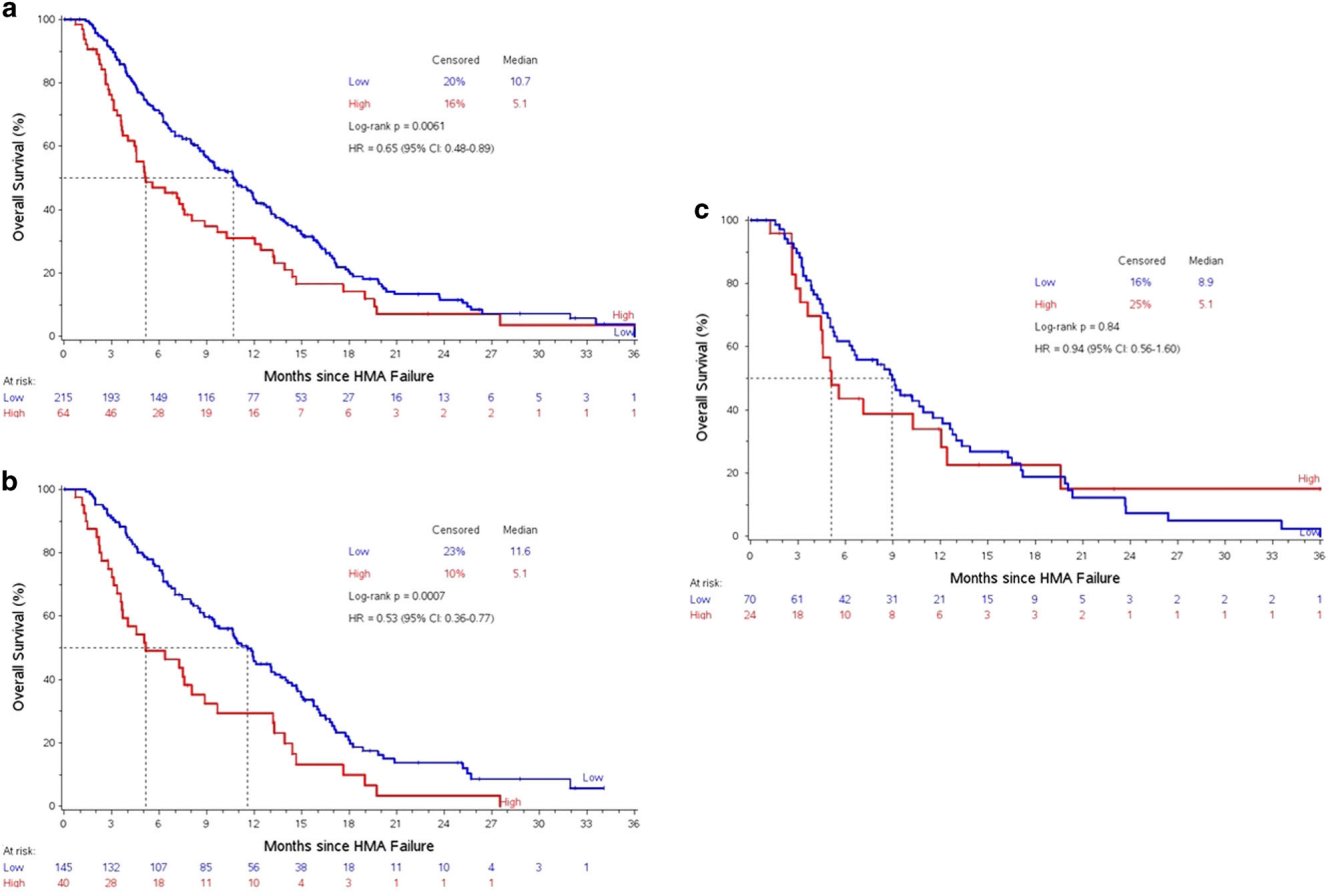
**a** Overall survival based on post-HMAF model, **b** overall survival based on post-HMAF model in the rigosertib arm, **c** overall survival based on post-HMAF model in the best supportive care arm

**Fig. 2:**
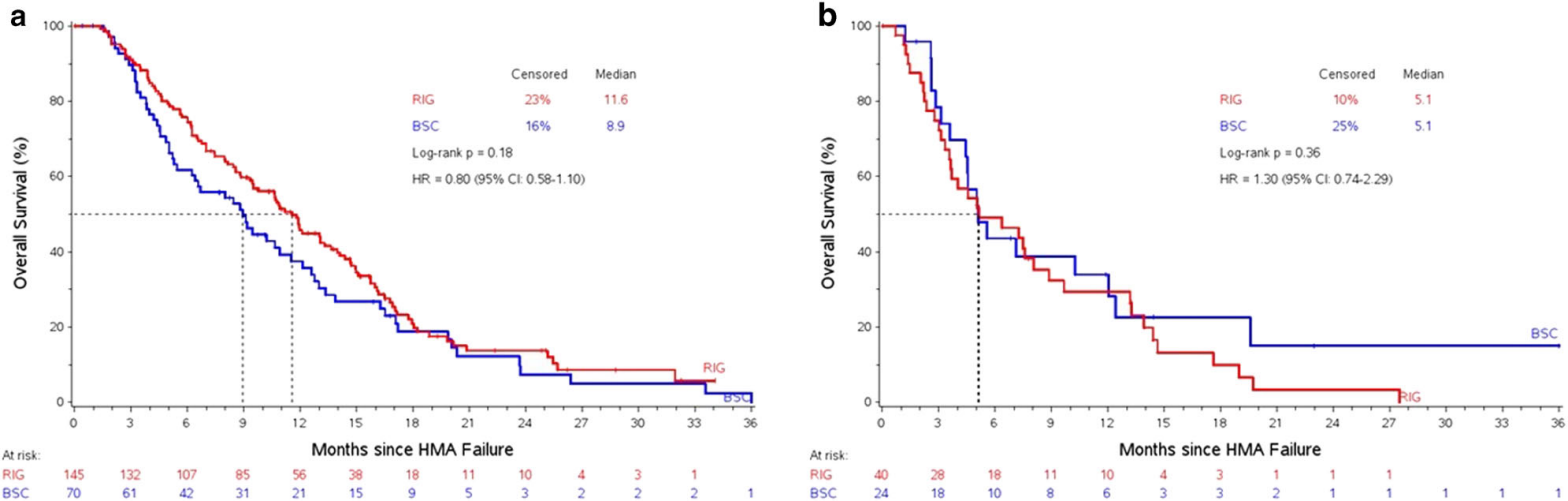
**a** Overall survival based on post-HMAF lower-risk category in patients treated with rigosertib vs. best supportive care, **b** overall survival based on post-HMAF higher-risk category in patients treated with rigosertib vs. best supportive care

Using the HMAF model, 215 patients (72%) had lower-risk disease: 145 treated with rigosertib and 40 treated with BSC, while 64 (21%) had higher-risk disease: 40 treated with rigosertib and 24 with BSC, respectively; 20 (7%) had missing data. The median OS for HMAF model lower-risk disease was 10.7 (95% CI 8.8–12.0) months, compared to 5.1 (95% CI 4.0–8.0) months for higher-risk (HR 0.65, 95% CI 0.48–0.89), *p* < 0.01 (Fig. 1a). The median OS for lower-risk vs. higher-risk patients treated with rigosertib was 11.6 (95% CI 9.5–13.3) vs. 5.1 (95% CI 3.6–8.9) months, *p* < 0.01 compared to 8.9 (95% CI 5.4–11.5) vs. 5.1 (95% CI 3.6–12.0) months for patients treated with BSC, *p* *<* 0.01 (Fig. 1b, c). Patients with lower-risk category who were treated with rigosertib had a median OS of 11.6 (95% CI 9.5–13.3) months compared to 8.9 (95% CI 5.4–11.5) months for patients treated with BSC, *p* = 0.18 (Fig. 2a) however, patients with higher-risk category had a similar median OS if they received rigosertib compared to BSC, 5.1 (95% CI 3.6–8.9) vs. 5.1 (95% CI 3.6–12.0) months, *p* = 0.36 (Fig. 2b).

While OS is generally poor in higher-risk MDS patients at the time of HMAF, there is heterogeneity even within this high-risk group. In this study, we confirm that the HMAF prognostic model is valid, and can accurately separate MDS patients into lower- and higher-risk disease at the time of HMAF in a patient population treated on a prospective clinical trial. To our knowledge this is the first such model to be validated in both retrospective HMA-treated cohorts and in a prospective randomized phase III trial. Risk stratifying patients who were enrolled on the trial by the new model demonstrated a median of 2.3 months OS benefit in the rigosertib arm compared to BSC, but this was not statistically significant as the study was not powered enough to detect such difference. This model may be used to risk stratify patients for clinical trial eligibility at the time of HMAF.
